# Synuclein-γ (SNCG) expression in ovarian cancer is associated with high-risk clinicopathologic disease

**DOI:** 10.1186/s13048-016-0281-4

**Published:** 2016-11-03

**Authors:** Anna Strohl, Kristina Mori, Stacey Akers, Wiam Bshara, Barbara Buttin, Peter J. Frederick, Irene B. Helenowski, Carl D Morrison, Kunle Odunsi, Julian C. Schink, Denise M. Scholtens, Jian-Jun Wei, J. Julie Kim

**Affiliations:** 1 Division of Gynecologic Oncology, Department of Obstetrics and Gynecology, Robert H. Lurie Comprehensive Cancer Center, Northwestern University Feinberg School of Medicine, Chicago, USA; 2 Division of Gynecologic Oncology, Roswell Park Cancer Institute, Buffalo, USA; 3 Department of Pathology, Roswell Park Cancer Institute, Buffalo, USA; 4 Gynecologic Oncology Program, Cadence Physician Group, Warrenville, USA; 5 Department of Preventive Medicine, Northwestern University Feinberg School of Medicine, Chicago, USA; 6 Department of Pathology, Robert H. Lurie Comprehensive Cancer Center, Feinberg School of Medicine, Northwestern University, 251 East Huron Street, Feinberg 7-334, Chicago, IL 60611 USA; 7 Division of Reproductive Science in Medicine, Department of Obstetrics and Gynecology, Robert H. Lurie Comprehensive Cancer Center, Northwestern University Feinberg School of Medicine, 303 E. Superior Street, 4-117, Chicago, IL 60611 USA

**Keywords:** Ovarian cancer, Synuclein gamma, High risk disease

## Abstract

**Background:**

Synuclein gamma (SNCG) expression is associated with advanced disease and chemoresistance in multiple solid tumors. Our goal was to determine if SNCG protein expression in ovarian cancer was correlated with clinicopathologic variables and patient outcomes.

**Methods:**

Tissue microarrays from primary tumors of 357 ovarian, fallopian tube, and primary peritoneal cancer patients, who underwent primary surgery at Roswell Park Cancer Institute between 1995 and 2007, were immunohistochemically stained for SNCG. A pathologist blinded to patient data scored tumors as positive if ≥10 % of the sample stained for SNCG. Medical records were reviewed for clinicopathologic and demographic variables. Between the positive and negative groups, Wilcoxon rank-sum test was used to compare the median ages and Fisher’s exact test was used to compare groups in categorical variables. Cox proportional hazard models examined associations between SNCG and overall and progression-free survival.

**Results:**

The median follow-up was 36 months, median overall survival was 39 months, and median progression-free survival was 18 months. SNCG presence was associated with clinical variables of serous histology, grade 3 disease, suboptimal debulking, ascites at surgery, FIGO stage III-IV cancer, or initial CA-125 level >485. There was no significant difference in overall survival (HR 1.06 95 % CI 0.81–1.39 P 0.69) or progression-free survival (HR 1.16 95 % CI 0.89–1.50 P 0.28) for patients with or without SNCG expression.

**Conclusions:**

SNCG expression in ovarian cancer is frequent in patients with high-risk features, but it does not correlate with chemotherapy response, overall survival, or progression-free survival.

**Electronic supplementary material:**

The online version of this article (doi:10.1186/s13048-016-0281-4) contains supplementary material, which is available to authorized users.

## Background

Ovarian cancer is a leading cause of cancer mortality in women, accounting for more deaths than any other gynecologic malignancy in the United States [1]. While overall cancer incidence and mortality rates for gynecologic malignancies have declined in the past decade, progress in ovarian cancer outcomes has been slow. Despite developments in cytotoxic chemotherapy, five-year survival rates for women diagnosed with ovarian cancer remain less than 50 % [2].

High rates of recurrence and associated mortality, coupled with advancements in the characterization of intracellular signaling pathways in carcinogenesis, have prompted investigation into other potential targets that can be used in the treatment of ovarian cancer, such as intracellular signaling pathways [3]. Among such targets, synuclein gamma (SNCG) was proposed as a potential target in ovarian cancer therapy [4].

Synucleins are a family of neuronal proteins expressed primarily in the peripheral nervous system. To date, three synuclein proteins have been identified: synuclein- α (SNCA), synuclein- β (SNCB), synuclein- γ (SNCG) [5, 6]. The former two, SNCA and SNCB, have been implicated in neurodegenerative disorders, such as Parkinson’s and Alzheimer’s disease [7, 8], while SNCG has been primarily linked with cancer. SNCG was first discovered in breast cancer tissue [9] and has since been detected in multiple solid tumors, including breast, lung, liver, esophagus, colon, bladder, pancreatic, and prostate cancers [10]. SNCG has also been identified in gynecologic processes, including benign pathology (endometriosis), as well as endometrial and ovarian cancers [10, 11]. SNCG expression occurs with advanced disease and chemoresistance in many cancers, and in breast cancer, SNCG has been causatively linked to increased proliferation, metastasis, and drug resistance [12].

Recent studies have demonstrated the role of synucleins as potential biomarkers in several cancer types, including ovarian cancer [13]. Several studies by immunohistochemistry (IHC) analysis demonstrated high levels of SNCG expression in up to 73 % of epithelial ovarian cancers [14–16] and one study showed that SNCG overexpression may promote the metastatic potential of ovarian cancer cells [17]. These findings suggest that SNCG may be a potential prognostic marker as well as a target for therapeutic drug development. Studies examining the correlation of SNCG expression with clinical outcomes are lacking, however, limited only to a single meta-analysis of gene expression profiles in ovarian cancer [18].

In this study, SNCG expression levels were examined using immunohistochemistry in primary and metastatic tumors of 357 patients with ovarian, fallopian tube, and primary peritoneal cancer. In particular, we examined the association of SNCG expression in ovarian cancer with worse outcomes, such as decreased progression free- and overall-survival and/or increased chemotherapy resistance.

## Methods

### Patient population

After obtaining IRB approval, the Pathology archive at Roswell Park Cancer Institute, Buffalo, New York was searched for ovarian, fallopian tube, and primary peritoneal cancer cases from 1995 to 2007. A chart review was conducted with extraction of clinical information, including patient’s age at the time of diagnosis, the surgical stage, postoperative treatment. All patients underwent a primary surgical staging surgery, including total abdominal hysterectomy with bilateral salpingo-oophorectomy, with or without pelvic and para-aortic lymph nodes dissection and pelvic washings, depending on tumor stage. Patients were treated according to National Comprehensive Cancer Network guidelines (https://www.nccn.org). Patient’s general information and tumor features are summarized in Table 1.

**Table 1:** Clinical and Pathologic Features of Patients

Variable	N (%)
No. of patients	357
Follow up time, months	
Median	36
Range	0–179
Age, year	
Median	63
Range	22–93
CA125 level	
≤ 485	141 (53.2)
> 485	124 (46.8)
Ascites	
No	73 (29.1)
Present	178 (70.9)
Optimal Debulking Surgery	
Optimal	245 (82.5)
Suboptimal	52 (17.5)
Stage	
I	33 (9.8)
II	31 (9.2)
III	234 (69.2)
IV	40 (11.8)
Primary Site of Disease	
Ovarian	300 (84.5)
Primary Peritoneal	53 (14.9)
Fallopian Tube	2 (0.6)
Histologic subtype	
Serous	262 (73.6)
Clear cell	22 (6.2)
Mucinous	12 (3.4)
Endometrioid	25 (7.0)
Other	35 (9.8)
Grade (FIGO)	
1	7 (2.6)
2	16 (6.1)
3	240 (91.3)
Recurrence	
No	64 (32.8)
Yes	131 (67.2)
SNCG Immunoexpression	
Negative	100 (28)
Positive	257 (72)
Status	
Alive, No evidence of disease (ANED)	67 (18.8)
Alive, with evidence of disease (AWED)	22 (6.2)
Died of Disease (DOD)	268 (75.0)
Survival Time (months)	
Median	39
95 % CI	34–44
Progression-free survival (months)	
Median	18
95 % CI	15–23

Some clinical information were missing from cases and thus the sum of cases for each feature listed may not equate to 357

### Histologic evaluation and high-density tissue microarray (TMA) preparation

Tumor grade was assessed using the International Federation of Gynecology and Obstetrics (FIGO) system and tumor stage was assigned based on the 2014 FIGO surgical staging guidelines. All slides were examined by a gynecologic pathologist for confirmation of tumor morphology and tumor grade. The viable tumor tissues and control tissues (fallopian tube) from each case were circled by pathologists. 0.6 mm tissue cores were punched and arrayed into high-density TMA receipting blocks.

### Immunohistochemistry

**Fig. 1:**
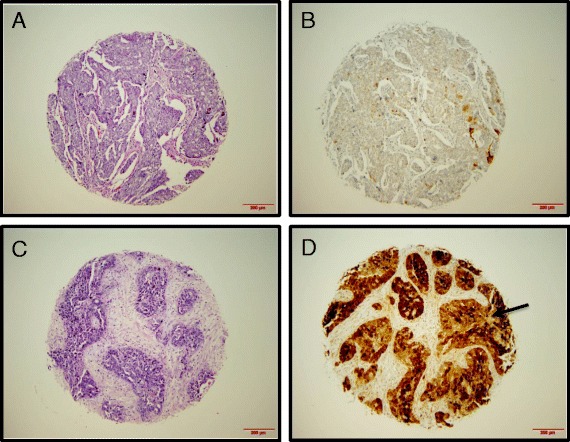
Immunohistochemical staining of SNCG in ovarian tumor sections in the TMA. Representative sections of negative **b** and positive **d** SNCG expression with corresponding H&E staining **a**, **c** are shown. Brown color (*arrow*) represents positive staining for SNCG

Tissues were prepared for analysis, as previously described by Mhawech-Fauceglia et al. [19]. Briefly, high-density TMA blocks were sectioned in 4 μm thickness followed by deparaffinization with xylene, then washed with ethanol. Sections were cooled for 20 min and incubated for 10 min with 3 % H2O2 to quench endogenous peroxidase activity. Blocking was performed using serum-free protein block, Dakocytomation (Carpenteria, CA) for 30 min. Antigen retrieval was done using a citrate-based buffer (pH 6; Bond Epitope Retrieval Solution, Leica Biosystems) and sections were incubated with the SNCG antibody (Abcam) for 1 h at room temperature on the Dako Autostainer Plus. The diaminobenzidine complex was used as a chromogen. Immunostained slides were blindly reviewed and scored by two gynecologic pathologists. Immunostain for SNCG was present in both the nucleus and cytoplasm. Immunoreactivity was semiquantitatively scored in immunointensity of 0 (negative), 1 + (weak), 2+ (moderate) and 3+ (strong) and immunopercentage of <10 %, 10–50 % and >50 %. For the sake of statistical analysis, tumors were grouped as positive (SNCG+) or negative (SNCG-). Tumors were considered SNCG+ if ≥10 % of the tumor epithelial cells were immunoreactive with immunointensity of ≥2. Examples of positive and negative cases are illustrated in Fig. 1a-d.

### Real time PCR

RNA from benign fallopian tube epithelium and ovarian cancer tissues was reverse transcribed. First-strand cDNA synthesis was performed using 700 ng of RNA and M-MLV reverse transcriptase (Life Technologies). Real time PCR was performed using Taqman reagent in a QuantStudio 5 system and primers to SNCG and the housekeeping gene TBP (Applied Biosystems/Life Technologies). Fold-change values were calculated using TBP as the housekeeping gene.

### Statistical analyses

To test the association between the biomarker and the clinical parameters, Wilcoxon rank-sum test was used to compare the median ages and Fisher’s exact test was used to compare frequencies and percentages for categorical variables. Progression-free survival (PFS), was defined as observed length of time from date of diagnosis to recurrence or censoring at date of last contact. Overall survival (OS) was defined as observed length of life from date of diagnosis to death or censoring at date of last contact. For survival analysis, Kaplan-Meier curves were used to estimate PFS, and OS curves. Curves were compared for tumors with and without SNCG expression using the log-rank test. Univariate cox proportional hazards models were used to estimate hazard ratios for SNCG status for both the OS and PFS outcomes. Multivariate Cox regression was then conducted with SNCG expression as the main predictor and adjustment for clinical factors that were associated with SNCG expression (CA125 level, ascites, debulking status, FIGO grade, tumor stage and histologic subtype). Since several of the clinical covariates were highly associated with each other, sensitivity of the hazard ratio estimates for SNCG was examined after adjustment for each clinical variable in separate models. All reported *p* values are two sided with *P* < 0.05 significance. Statistical analyses was performed using SAS v 9.4. Unpaired *t*-test was used to compare SNCG mRNA levels in benign fallopian tube and ovarian cancer tissues using Graphpad Prism version 6.0 (Graphpad Software, La Jolla, CA, USA).

## Results

### Patient characteristics

The clinical and pathologic features of 357 patients with primary ovarian, fallopian tube, and primary peritoneal cancer are summarized in Table 1. The mean age at diagnosis was 63 years. All patients (*n* = 357) underwent primary surgery with 82.5 % optimal debulking. No patients received neoadjuvant chemotherapy or radiation therapy. The majority of patients had advanced stage (FIGO Stage III/IV: 81.1 %), were serous type (73.6 %) and high-grade (FIGO Grade 3: 91.3 %) disease. About 70 % of patients had preoperative ascites and 46.8 % had CA-125 levels > 485 U/mL at the time of surgery. Median follow-up time was 36 months (range 0–179 months). At the time of data analysis, 67.2 % of patients had experienced disease recurrence and 24.9 % were surviving.

### Clinical characteristics and SNCG expression

**Fig. 2:**
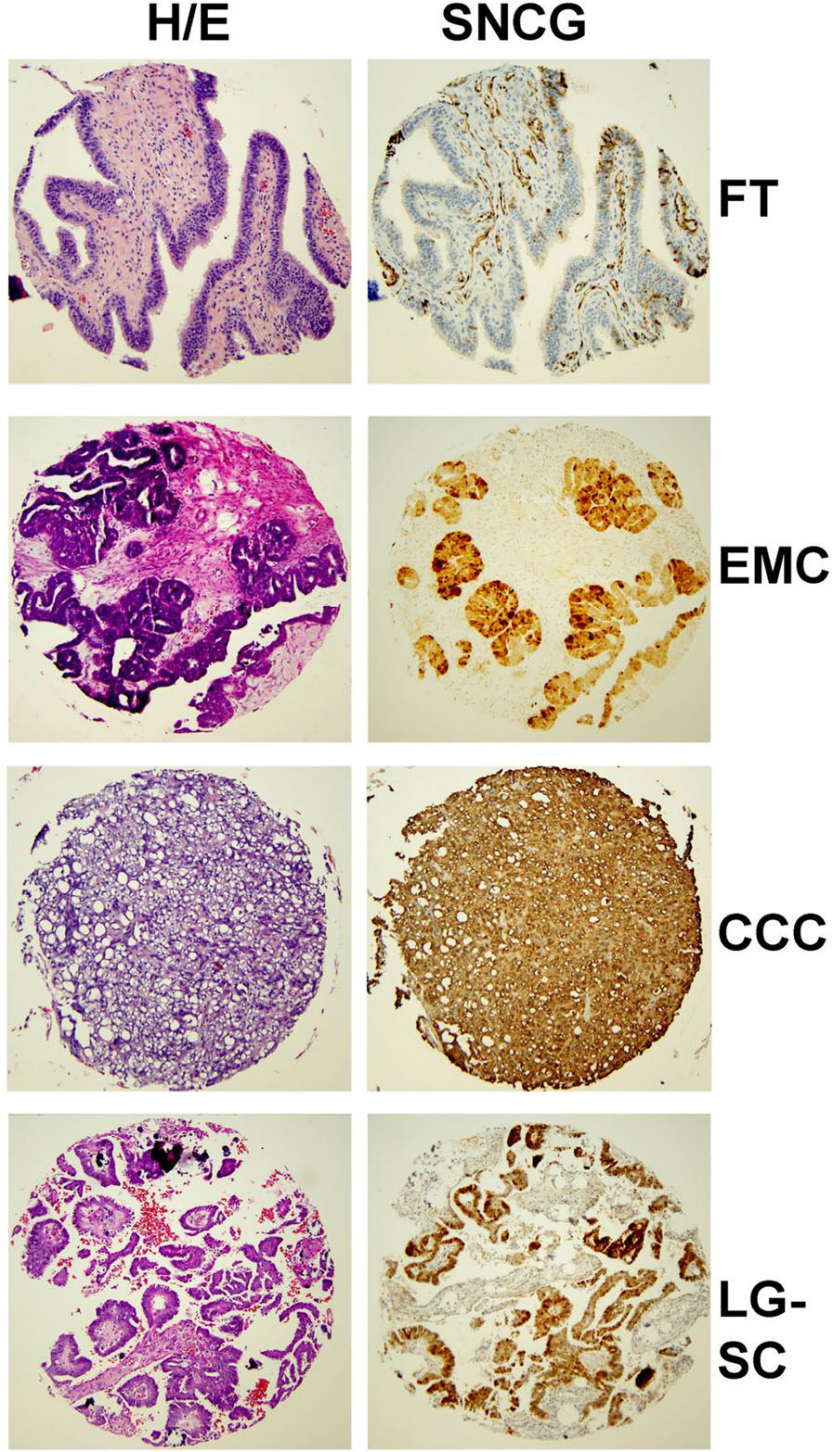
Immunohistochemical staining of SNCG in different ovarian tumor types in the TMA. Representative sections of H&E that are SNCG positive are shown FT: fallopian tube, EMC: endometrioid carcinoma, CCC: clear cell carcinoma, LG-SC: low grade serous carcinoma. Brown color represents positive staining for SNCG. Each core is 1 mm in diameter

SNCG expression was positive in 72 % (257/357) of primary tumors. Examples of positive and negative cases are illustrated in Fig. 1a-d. SNCG was expressed in different types of ovarian cancers as well, including fallopian tube, endometrioid carcinoma, clear cell carcinoma, and low grade serous carcinoma (Fig. 2). SNCG overexpression was associated with tumor type, stage, grade and other clinical parameters (Table 2). SNCG overexpression was significantly higher in cases with serous histology than in other histologic variants (*p* < 0.0001). In 212 cases with serous histology, 85 % of cases were SNCG positive while only 43 % of other types were SNCG positive. Similarly, SNCG overexpression was much more common in high grade tumors (73 %) than in low grade (47 %) (*p* = 0.01). Stage III/IV disease had significantly high SNCG expression in comparison to stage I/II (*p* < 0.0001). About 88 % of suboptimal debulking tumors showed SNCG overexpression in comparison to 69 % of optimal debulking cases (*p* = 0.004). Patients with ascites also showed significantly higher SNCG expression than those without ascites (*p* = 0.01). When dividing CA-125 scores of below and above 485, SNCG expression was marginally associated with high CA-125 expression as well (*p* = 0.02). There was no association between age at diagnosis or site of primary disease and SNCG expression.

**Table 2:** Clinical Factors by SNCG expression status

	SNCG positive	SNCG negative	*p*-value
No. of patients (n = 357)	257 (72 %)	100 (28 %)	
Age, year			
Median	63.0	63.5	0.93
Range	22–91	36–93	
CA125 level			
≤ 485	93 (48.7 %)	48 (64.9 %)	0.02
> 485	98 (51.3 %)	26 (35.1 %)	
Ascites			
No	44 (24.4 %)	29 (40.8 %)	0.01
Present	136 (75.6 %)	42 (59.2 %)	
Debulking Status			
Optimal	169 (78.6 %)	76 (92.7 %)	0.004
Suboptimal	46 (21.4 %)	6 (7.3 %)	
Stage			
I/II	30 (12.2 %)	34 (36.6 %)	<0.0001
III/IV	215 (87.8 %)	59 (63.4 %)	
Primary Site of Disease			
Ovarian	219 (85.9 %)	81 (81.0 %)	0.28
Primary Peritoneal	34 (13.3 %)	19 (19.0 %)	
Fallopian Tube	2 (0.8 %)	0 (0.0 %)	
Histologic subtype			
Serous	181 (82.7 %)	31 (38.3 %)	<0.0001
Non-serous	38 (17.3 %)	50 (61.7 %)	
Grade (FIGO)			
1/2	11 (5.8 %)	12 (16.0 %)	0.01
3	177 (94.2 %)	63 (84.0 %)	
Recurrence			
No	23 (19.49 %)	15 (29.41 %)	0.17
Yes	95 (80.51 %)	36 (70.59 %)	
Status			
Alive	60 (23.3 %)	29 (29.0 %)	0.28
Dead	197 (76.7 %)	71 (71.0 %)	

Percentages are based on denominators of available data due to missing information from cases

To provide a quantitative measure of SNCG levels using real time PCR, RNA from tissue samples outside of the cases used for the tissue microarray, was analyzed. SNCG expression from benign human fallopian tube epithelium and ovarian cancer tissues was compared. Benign fallopian tube epithelium expressed less SNCG than ovarian cancer tissues (Additional file 1: Figure S1), which supports the immunohistochemical analysis of SNCG (Fig. 2).

### Survival analyses

**Fig. 3:**
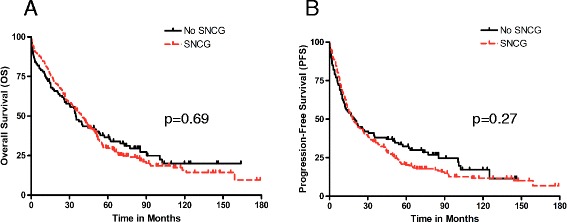
Kaplan–Meier survival curves for patients with ovarian cancer stratified according to SNCG expression (positive versus negative). Curves for **a** overall survival and **b** progression-free survival are shown

The median OS for the entire study group was 39 months (95 % CI: 34–44 months) and 5-years OS was 31.57 %. Median progression-free survival (PFS) was 18 months (95 % CI: 15–23). There was no significant difference in OS (HR 1.06; 95 % CI 0.81–1.39, *p* = 0.69) or PFS (HR 1.16; 95 % CI 0.89–1.50, *p* = 0.28) for patients with SNCG+ compared to those with SNCG- tumors (Fig. 3). The lack of association between SNCG expression and OS and PFS persisted after adjustment for all clinical variables associated with SNCG (Additional file 2: Table S1).

### Metastatic disease

We further explored SNCG expression in metastases. Among patients with metastatic tumor samples (*n* = 184), 153 (83 %) of metastatic tumors demonstrated SNCG expression. The presence of SNCG expression was compared between primary and metastatic tumors within the same patient: 12 (6.6 %) metastatic tumors gained expression of SNCG, 34 (18.6 %) lost expression, 119 (64 %) maintained expression, and 19 (10.4 %) never demonstrated SNCG expression (Table 3). The 5-years OS by groups were: 41.67 % for tumors gained expression of SNCG, 32.09 % for lost expression, 20.59 % for maintained expression, and 21.05 % never demonstrated SNCG expression. There was no significant difference in OS among patients in whom primary tumors gained SNCG expression, lost SNCG expression, maintained SNCG expression, or never expressed SNCG (log-rank *p* = 0.23).

**Table 3:** SNCG Expression in Metastases of Primary Tumors

Primary/Metastasis	N	N event	Median OS (months)	2 years OS	5 years OS
−/+ (Gained expression)	12	10	47 (30,102)	91.67 %	41.67 %
+/− (Lost expression)	34	26	32 (17,55)	58.82 %	32.09 %
+/+ (Maintained expression)	118	104	39 (27,44)	65.25 %	20.59 %
−/− (Never expressed)	19	17	17 (9,44)	47.37 %	21.05 %
Log-rank *p* = 0.23					
Combined Metastatic Expression					
Positive	130	114	40 (29,44)	67.69 %	22.65 %
Negative	53	43	29 (15,44)	54.72 %	28.18 %

### SNCG expression and chemotherapy response/platinum resistance

We further explored the impact of SNCG on chemotherapy response. Table 4 demonstrates the distribution of SNCG expression by platinum-refractory (defined as disease progression with first-line chemotherapy) or platinum-sensitive (defined as disease recurrence within 6 months of first-line chemotherapy) disease status compared to those patients with no evidence of disease (NED). Results show a marginally greater prevalence of platinum-refractory cases in cases with positive SNCG expression (*p* = 0.08). There was no significant difference in SNCG status in patients with platinum-sensitive disease (*p* = 0.79).

**Table 4:** SNCG expression in response to chemotherapy. Comparisons between patients with platinum-refractory (defined as disease progression with first-line chemotherapy) and patients with no evidence of disease (NED) or platinum-sensitive disease status (defined as disease recurrence within 6 months of first-line chemotherapy) and platinum-resistant are shown

SNCG Expression	Platinum-refractory (*N* = 107)	NED (*N* = 164)	*p*-value
No	20 (18.7 %)	46 (28.0 %)	0.08
Yes	87 (81.3 %)	118 (72.0 %)	
SNCG Expression	Platinum sensitive (*N* = 145)	Platinum resistant (*N* = 19)	
No	40 (27.6 %)	6 (31.6 %)	0.79
Yes	105 (72.4 %)	13 (68.4 %)	

## Discussion

This study is the first to formally evaluate the proposed association between SNCG protein expression and clinical outcomes in patients with ovarian, fallopian tube, and primary peritoneal cancers. SNCG is a new potential biomarker and demonstrates to be specific and reliable for immunohistochemistry in formalin-fixed and paraffin-embedded tissue sections. SNCG has been examined in several different tumor types [10, 13–16]. Although the cut-off value of immunoreactivity for positive and negative SNCG has not been established for each tumor type, we found it was meaningful and reproducible in ovarian cancer when 10 % of tumor cells have moderate to strong immunoreactivity for SNCG. In our series of 357 patients, expression of SNCG was identified in 72 % of primary tumors, a percentage similar to the 73 % expression that has previously been reported in ovarian carcinomas [14]. The hypothesis, namely that SNCG expression was associated with worse clinical outcomes, was refuted, as the results of our study found no association between SNCG expression and OS or PFS in primary tumors.

While SNCG expression did not correlate with clinical outcomes such as PFS, OS, or chemoresistance, the results of our study did find that there was a significant association between SNCG expression and high-risk clinicopathological factors, such as serous histology, high grade disease, advanced stage, and suboptimal debulking surgery. These associations suggest that while SNCG expression might not be a single prognostic marker, it may be an indicator for high-risk disease and may play a role in the pathogenesis of disease progression. Furthermore, SNCG expression may provide additional evidence of disease burden, given its association with advanced high-grade disease. Given its potential as a marker for high-risk disease, future studies should focus on the potential role of secretory SNCG a surrogate marker for disease burden, such as CA125. Prior studies have demonstrated that SNCG is detectable in the serum of patients with cancer [20–23]. The next direction would be to test serum samples from patients that were collected at the same time as tumor tissue in order to correlate serum and tissue SNCG levels.

In regards to SNCG expression in metastatic ovarian cancer, our data did not demonstrate a significant difference in OS among patients in whom primary tumors gained or lost SNCG expression; however, our data did demonstrate that the majority of metastatic tumors (83 %) demonstrated SNCG expression. This data supports prior studies that implicate SNCG overexpression may promote the metastatic potential of ovarian cancer cells [17] and further supports the association of SNCG expression with high-risk clinicopathologic disease. Our findings of high levels of SNCG expression in ovarian cancer were consistent with SNCG expression in multiple cancer types, including breast, liver, prostate, and colon cancer [14, 21, 24, 25]. However, unlike breast [12] and endometrial cancers [19] in which high SNCG expression has been correlated with adverse clinical outcomes, our study found no correlation between high SNCG expression and clinical outcomes.

There are several possibilities for the lack of association with OS or PFS in this study in ovarian cancer. First, the study may have been underpowered to detect a significant difference in clinical outcomes. The association of SNCG expression with high-risk disease would suggest that there is a difference in clinical outcomes such as PFS or OS, however this was not seen in our cohort. Next, ovarian cancer is a heterogeneous cancer with multiple histologic subtypes and underlying molecular aberrations [26] and thus SNCG expression may influence tumor phenotype differently depending on co-existing histologic and molecular factors. Furthermore, as SNCG expression is not specific to ovarian cancer, its function in ovarian cancer may be a feature of malignancy in general. Finally, we evaluated the presence of SNCG, not its function, nor its level of expression. Tumors may change such genomic outcomes and our study is insufficient to determine either the underlying somatic or germline polymorphisms or the epigenetic actions on SNCG genes. Future studies might focus on the correlation of SNCG protein and gene expression in combination with other markers of disease, such as CA125 levels.

In conclusion, this study found that while SNCG expression is often present in ovarian carcinoma, the positive or negative expression of SNCG protein alone is not independently associated with clinical prognosis. Our results did indicate that SNCG expression is associated with clinicopathologic features of high-risk disease, suggesting that SNCG expression may play a role in severity of disease and be a marker for aggressive disease. Furthermore, as targeted drug therapy develops, recognition of SNGC expression in ovarian cancer will remain important as we continue to discover ways to improve outcomes in patients diagnosed with this deadly disease.

## Conclusions

Expression of SNCG is associated with clinicopathologic variables of aggressive and advanced disease but not with overall survival or progression free survival. SNCG may serve as a novel biomarker for aggressive or advanced ovarian carcinoma and warrants further investigation to determine its role in this disease.

## Additional files

Additional file 1: Figure S1. Expression of SNCG mRNA in benign fallopian tube epithelium and ovarian cancer tissues. RNA from human benign fallopian tube epithelium and ovarian cancer tissues was subjected to RT-real time PCR for SNCG. SNCG as a ratio of the housekeeping gene TBP is presented using delta delta CT calculations. * = *p* < 0.05. FT – fallopian tube; OvCa – ovarian cancer. (PPTX 64 kb)
Additional file 2: Table S1. Hazard ratios (HR) from multivariate Cox regression models for PFS and OS involving SNCG expression and adjusting for relevant demographic and clinical factors. (DOCX 123 kb)

## Abbreviations

CA-125: Cancer antigen-125
FIGO: International Federation of Gynecology and Obstetrics
HR: Hazard ratio
NED: No evidence of disease
OS: Overall survival
PFS: Progression-free survival
SNCA: Synuclein-alpha
SNCB: Synuclein-beta
SNCG: Synuclein gamma
TMA: Tissue microarray
U/mL: Units per milliliter
Yr: Year
