# Supplementary material for: Synuclein-γ (SNCG) expression in ovarian cancer is associated with high-risk clinicopathologic disease
Source: J Ovarian Res. 2016 Nov 3;9:75. doi: 10.1186/s13048-016-0281-4 (PMC5094138; doi:10.1186/s13048-016-0281-4)
Supplement: Additional file 1: Figure S1. — Expression of SNCG mRNA in benign fallopian tube epithelium and ovarian cancer tissues. RNA from human benign fallopian tube epithelium and ovarian cancer tissues was subjected to RT-real time PCR for SNCG. SNCG as a ratio of the housekeeping gene TBP is presented using delta delta CT calculations. * = p < 0.05. FT – fallopian tube; OvCa – ovarian cancer. (PPTX 64 kb) [file 13048_2016_281_MOESM1_ESM.pptx]

## Slide 1
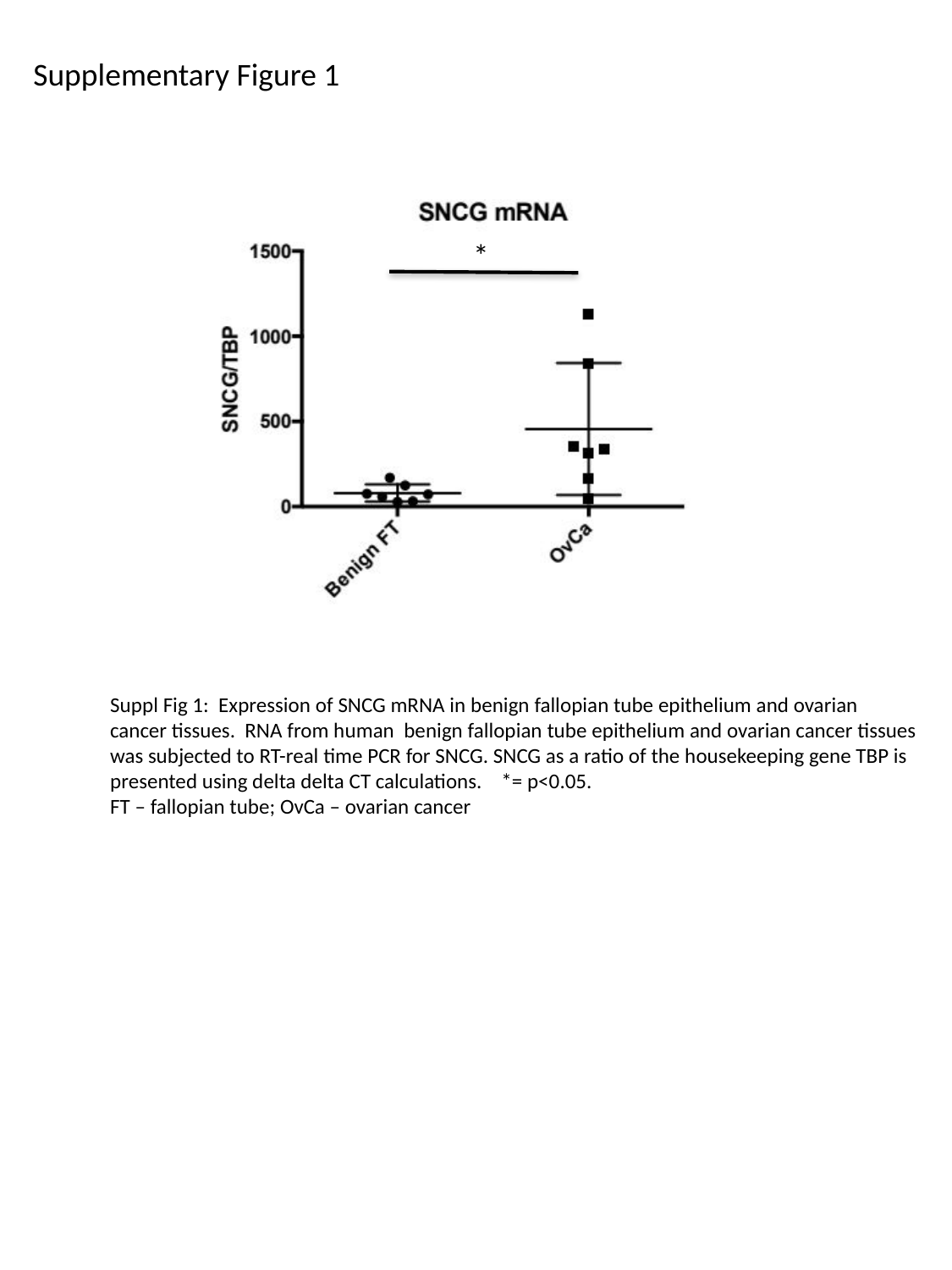

Supplementary Figure 1
*
Suppl Fig 1: Expression of SNCG mRNA in benign fallopian tube epithelium and ovarian cancer tissues. RNA from human benign fallopian tube epithelium and ovarian cancer tissues was subjected to RT-real time PCR for SNCG. SNCG as a ratio of the housekeeping gene TBP is presented using delta delta CT calculations. *= p<0.05.
FT – fallopian tube; OvCa – ovarian cancer
