# Supplementary material for: Synuclein-γ (SNCG) expression in ovarian cancer is associated with high-risk clinicopathologic disease
Source: J Ovarian Res. 2016 Nov 3;9:75. doi: 10.1186/s13048-016-0281-4 (PMC5094138; doi:10.1186/s13048-016-0281-4)
Supplement: Additional file 2: Table S1. — Hazard ratios (HR) from multivariate Cox regression models for PFS and OS involving SNCG expression and adjusting for relevant demographic and clinical factors. (DOCX 123 kb) [file 13048_2016_281_MOESM2_ESM.docx]

**Supplemental Table 1:** Hazard ratios (HR) from multivariate Cox regression models for PFS and OS involving SNCG expression and adjusting for relevant demographic and clinical factors.

| Variable | Outcome | HR | 95% lower limit | 95% upper limit | p-value | Outcome | HR | 95% lower limit | 95% upper limit | p-value |
| --- | --- | --- | --- | --- | --- | --- | --- | --- | --- | --- |
| SNCG | PFS | 1.032 | 0.755 | 1.411 | 0.84 | OS | 0.887 | 0.638 | 1.231 | 0.47 |
| > CA-125 median | PFS | 1.486 | 1.129 | 1.956 | 0.005 | OS | 1.535 | 1.146 | 2.057 | 0.004 |
|  |  |  |  |  |  |  |  |  |  |  |
| SNCG | PFS | 1.204 | 0.866 | 1.674 | 0.27 | OS | 0.996 | 0.707 | 1.404 | 0.98 |
| Ascites | PFS | 1.841 | 1.312 | 2.582 | 0.0004 | OS | 1.785 | 1.241 | 2.568 | 0.002 |
|  |  |  |  |  |  |  |  |  |  |  |
| SNCG | PFS | 1.166 | 0.863 | 1.575 | 0.32 | OS | 1.004 | 0.735 | 1.372 | 0.98 |
| Optimal bulking | PFS | 0.609 | 0.440 | 0.842 | 0.003 | OS | 0.530 | 0.378 | 0.743 | 0.0002 |
|  |  |  |  |  |  |  |  |  |  |  |
| SNCG | PFS | 1.026 | 0.754 | 1.395 | 0.87 | OS | 0.859 | 0.624 | 1.181 | 0.35 |
| Grade 3 vs. 1/2 | PFS | 2.263 | 1.251 | 4.092 | 0.007 | OS | 2.484 | 1.302 | 4.740 | 0.006 |
|  |  |  |  |  |  |  |  |  |  |  |
| SNCG | PFS | 1.075 | 0.776 | 1.490 | 0.66 | OS | 0.998 | 0.714 | 1.394 | 0.99 |
| Serous histology | PFS | 1.383 | 0.999 | 1.916 | 0.051 | OS | 1.213 | 0.866 | 1.698 | 0.26 |
|  |  |  |  |  |  |  |  |  |  |  |
| SNCG | PFS | 0.854 | 0.645 | 1.130 | 0.27 | OS | 0.751 | 0.562 | 1.003 | 0.052 |
| Stage III/IV vs. I/II | PFS | 3.254 | 2.183 | 4.851 | <.0001 | OS | 3.040 | 1.983 | 4.660 | <.0001 |
